# Epigenetic regulation of lung cancer cell proliferation and migration by the chromatin remodeling protein BRG1

**DOI:** 10.1038/s41389-019-0174-7

**Published:** 2019-11-06

**Authors:** Zilong Li, Jun Xia, Mingming Fang, Yong Xu

**Affiliations:** 1 Key Laboratory of Targeted Intervention of Cardiovascular Disease and Collaborative Innovation Center for Cardiovascular Translational Medicine, Department of Pathophysiology, Nanjing Medical University, Nanjing, China; 2 Department of Respiratory Medicine, The Affiliated Hospital of Nanjing University of Chinese Medicine, Jiangsu Province Hospital of Traditional Chinese Medicine, Nanjing, China; 3 Department of Clinical Medicine and Laboratory Center for Experimental Medicine, Jiangsu Health Vocational College, Nanjing, China; 4 Institute of Biomedical Research, Liaocheng University, Liaocheng, China

**Keywords:** Epigenetics, Chromatin, Cell growth, Cell migration

## Abstract

Malignant lung cancer cells are characterized by uncontrolled proliferation and migration. Aberrant lung cancer cell proliferation and migration are programmed by altered cancer transcriptome. The underlying epigenetic mechanism is unclear. Here we report that expression levels of BRG1, a chromatin remodeling protein, were significantly up-regulated in human lung cancer biopsy specimens of higher malignancy grades compared to those of lower grades. Small interfering RNA mediated depletion or pharmaceutical inhibition of BRG1 suppressed proliferation and migration of lung cancer cells. BRG1 depletion or inhibition was paralleled by down-regulation of cyclin B1 (CCNB1) and latent TGF-β binding protein 2 (LTBP2) in lung cancer cells. Further analysis revealed that BRG1 directly bound to the CCNB1 promoter to activate transcription in response to hypoxia stimulation by interacting with E2F1. On the other hand, BRG1 interacted with Sp1 to activate LTBP2 transcription. Mechanistically, BRG1 regulated CCNB1 and LTBP2 transcription by altering histone modifications on target promoters. Specifically, BRG1 recruited KDM3A, a histone H3K9 demethylase, to remove dimethyl H3K9 from target gene promoters thereby activating transcription. KDM3A knockdown achieved equivalent effects as BRG1 silencing by diminishing lung cancer proliferation and migration. Of interest, BRG1 directly activated KDM3A transcription by forming a complex with HIF-1α. In conclusion, our data unveil a novel epigenetic mechanism whereby malignant lung cancer cells acquired heightened ability to proliferate and migrate. Targeting BRG1 may yield effective interventional strategies against malignant lung cancers.

## Introduction

Lung cancer represents one of the most deadly cancers in both industrialized and developing countries^1,2^. During lung cancer oncogenesis, normal epithelial cells acquire the ability of aggressive proliferation and migration contributing to tumor growth and dissemination^3^. Aberrant cancer cell proliferation and migration are regulated by a myriad of signaling pathways, which converge in the nucleus to re-program the cellular transcriptome. Typically, genes that promote cell proliferation and/or migration are up-regulated in lung cancer cells compared to the normal cells. For instance, hypoxia, a characteristic feature of the tumor microenvironment, promotes lung cancer cell proliferation and migration by activating the expression of genes involved in cell cycling (e.g., cyclins), epithelial-to-mesenchymal transition (e.g., Twist), glucose metabolism (e.g., Glut1), cell survival and apoptosis (e.g., Bcl2), and angiogenesis (e.g., VEGF)^4^. Hypoxia-inducible factor (HIF) family of transcription factors are considered the primary mediator of the cellular response to hypoxia in lung cancer cells although other transcription factors may also play critical roles^5^. The epigenetic mechanism whereby hypoxia skews lung cancer cell transcriptome to promote proliferation and migration is not completely understood.

Brahma related gene 1 (BRG1) is the catalytic component of the mammalian SWI/SNF chromatin remodeling complex intimately involved in transcriptional regulation^6^. BRG1 characteristically functions within the confines of a multifactor epigenetic complex that include not only other subunits of the SWI/SNF proteins (e.g., BRG1-associated factors or BAFs) but histone/DNA modifying enzymes and non-coding regulatory RNAs^7^. The compositions of the BRG1-containing complexes appear to be rather flexible so that BRG1 regulates transcription in a cell type and context-dependent manner. BRG1 is essential for embryogenesis as evidenced by the observation that germline deletion of BRG1 in mice causes developmental arrest and lethality^8^. Postnatal activation of BRG1, however, has been found to be associated with a host of human diseases including atherosclerosis^9^, pulmonary hypertension^10^, pathological hypertrophy^11^, alcoholic steatohepatitis^12,13^, and abdominal aortic aneurysm^14^.

The relationship between BRG1 and lung carcinogenesis remains controversial with evidence arguing both for and against BRG1 being a promoter of lung cancer development and progression. On the one hand, loss-of-function mutations of BRG1 have been frequently identified in lung cancer cells^15^. In addition, BRG1 inactivation is associated with increased lung cancer aggressiveness in humans^16^, which seems to suggest that BRG1 may function as a suppressor of lung oncogenesis. In contrast, loss of BRG1 sensitizes non-small cell lung cancer to chemotherapeutic drugs targeting either CDK4/6^17^ or aurora kinase A^18^. In the present study, we investigated the mechanism whereby BRG1 regulates hypoxia-induced proliferation and migration of lung cancer cells. We report that BRG1 contributes to hypoxia-induced transcription of genes involved in cancer cell proliferation and migration by interacting with different sequence-specific transcription factors. BRG1 activates transcription in lung cancer cells by recruiting the demethylase KDM3A and by directly up-regulating KDM3A expression. Therefore, targeting BRG1 may yield effective interventional strategies against malignant lung cancers.

## Results

### Elevated BRG1 expression correlates with augmented lung cancer malignancy in humans

**Fig. 1:**
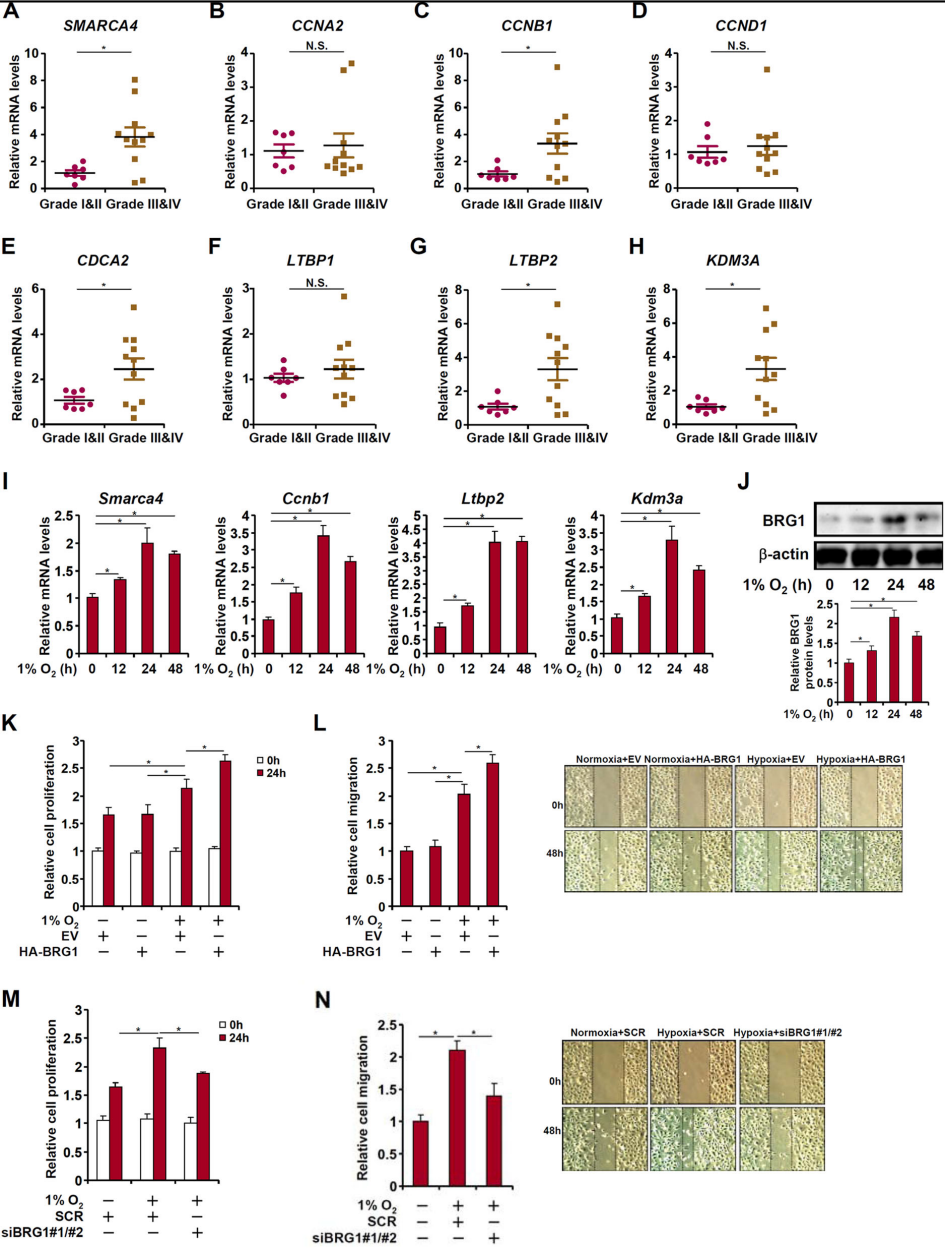
BRG1 regulates lung cancer cell proliferation and migration. **a**–**h** Gene expression levels in human lung cancer specimens are examined by qPCR. **i**, **j** LLC cells were exposed to 1% O_2_ and harvested at indicated time points. Gene expression levels were examined by qPCR and Western. **k**, **l** LLC cells were transfected with HA-tagged BRG1 or an empty vector (EV) before exposure to 1% O_2_ for 24 h. MTT assay and wound healing assay were performed and quantified as described in Methods. **m**, **n** LLC cells were transfected with siRNA targeting BRG1 or scrambled siRNA (SCR) before exposure to 1% O_2_ for 24 h. MTT assay and wound healing assay were performed and quantified as described in Methods

We first examined the expression levels of BRG1 in a small cohort of lung cancer biopsy specimens. Compared to those of low malignancies (grade I and grade II), highly malignant tumors (grade III and grade IV) exhibit much higher expression of BRG1 (*SMARCA4*, Fig. 1a). We also compared the expression of genes involved in cell proliferation and migration between the two groups. Whereas cyclin B1 (*CCNB1*, Fig. 1c), cell division cycle associated 2 (*CDCA2*, Fig. 1e), and latent TGF binding protein 2 (*LTBP2*, Fig. 1g) were up-regulated in malignant lung cancers, cyclin A1 (*CCNA1*, Fig. 1b), cyclin D1 (*CCND1*, Fig. 1d), and latent TGF binding protein 1 (*LTBP1*, Fig. 1f) were not significantly altered. We then exposed lung cancer cells (LLC) to the treatment of low oxygen tension (hypoxia), which is known to promote cancer cell proliferation and migration^19,20^. Hypoxia exposure stimulated BRG1 expression in a time course dependent manner at both mRNA (Fig. 1i) and protein (Fig. 1j) levels.

### BRG1 regulates lung cancer cell proliferation and migration

To examine whether BRG1 could regulate hypoxia-induced proliferation and migration of lung cancer cells, the following experiments were performed. MTT assay and wound healing assay showed that exposure to 1% O2 augmented proliferation and migration of LLC cells by ~35 and 110%, respectively; over-expression of BRG1 further enhanced hypoxia-induced LLC proliferation (Fig. 1k) and migration (Fig. 1l). Of note, BRG1 over-expression by itself did not significantly alter proliferation (Fig. 1k) or migration (Fig. 1l) suggesting that the ability of BRG1 to regulate cancer cell behavior may require a specific pro-malignancy stimulus (e.g., hypoxia). On the contrary, depletion of BRG1 by siRNA abolished hypoxia-induced proliferation (Fig. 1m) and migration (Fig. 1n) of LLC cells.

### BRG1 is essential for hypoxia-induced expression of cyclin B1 and latent TGF binding protein 2 in lung cancer cells

**Fig. 2:**
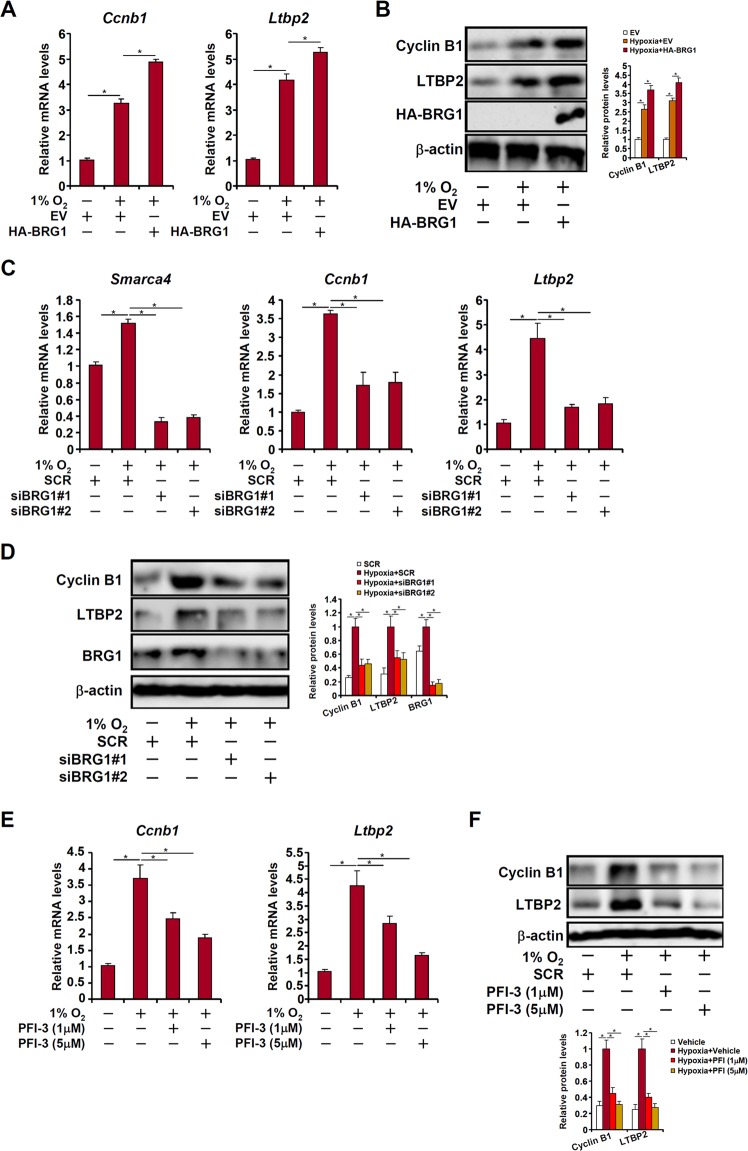
BRG1 is essential for hypoxia-induced expression of cyclin B1 and latent TGF binding protein 2 in lung cancer cells. **a**, **b** LLC cells were transfected with HA-tagged BRG1 or an empty vector (EV) before exposure to 1% O_2_ for 24 h. Gene expression levels were examined by qPCR and Western. **c**, **d** LLC cells were transfected with siRNA targeting BRG1 or SCR before exposure to 1% O_2_ for 24 h. Gene expression levels were examined by qPCR and Western. **e**, **f** LLC cells were treated with a BRG1 inhibitor and exposed to 1% O_2_ for 24 h. Gene expression levels were examined by qPCR and Western

The observations that there was a concurrent up-regulation of BRG1, CCNB1, and LTBP2 in high-grade human lung cancers combined with the well-established roles of CCNB1 and LTBP2 in cancer cell proliferation and migration prompted us to investigate whether BRG1 may contribute to hypoxia-induced LLC cell proliferation and migration by regulating CCNB1 and LTBP2 expression. As shown in Fig. 2a, b, over-expression of BRG1 augmented hypoxia-induced expression of CCNB1 and LTBP2. In contrast, depletion of BRG1 by two separate pairs of siRNAs dampened induction of CCNB1 and LTBP2 by hypoxia in LLC cells (Fig. 2c, d). In addition, treatment with a specific BRG1 inhibitor PFI-3 dose-dependently suppressed hypoxia-induced expression of CCNB1 and LTBP2 (Fig. 2e, f).

### BRG1 regulates CCNB1 transcription in lung cancer cells

**Fig. 3:**
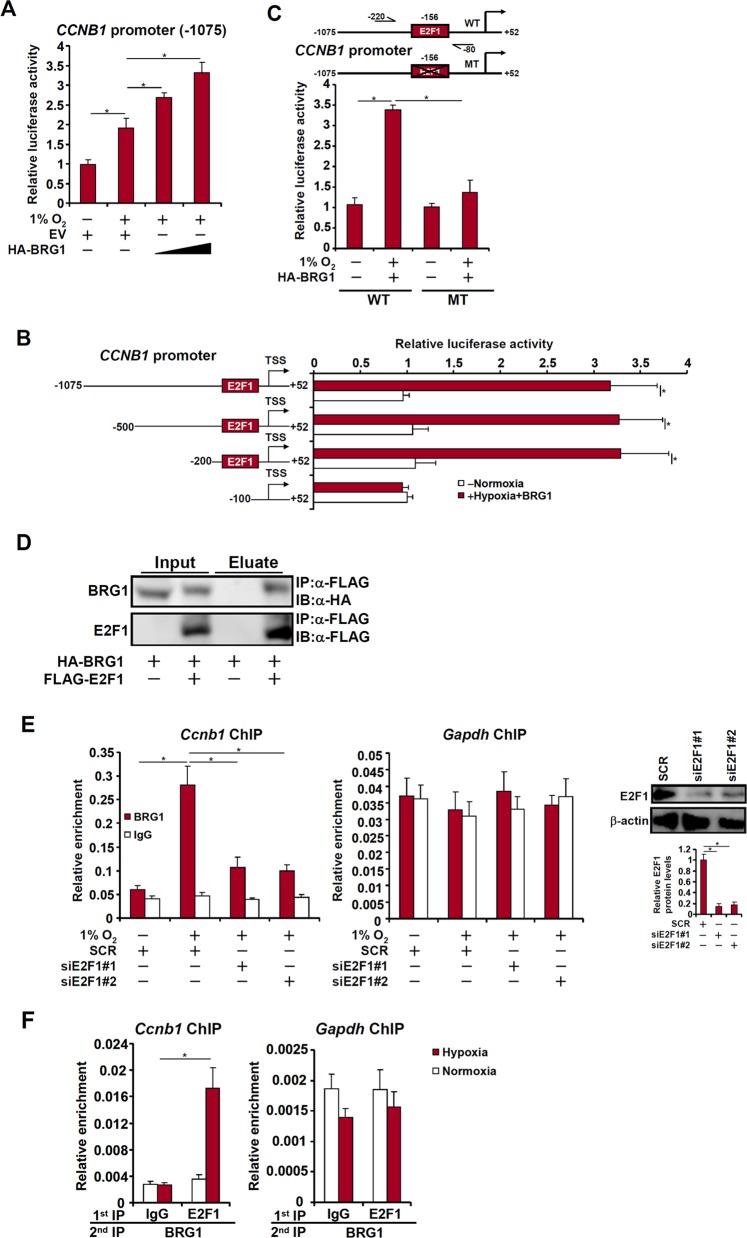
BRG1 regulates CCNB1 transcription in lung cancer cells. **a** A CCNB1 promoter-luciferase construct was transfected into LLC cells with or without BRG1 followed by exposure to 1% O_2_ for 24 h. Luciferase activities were normalized by both protein concentration and GFP fluorescence. **b** CCNB1 promoter-luciferase constructs of different lengths were transfected into LLC cells with or without BRG1 followed by exposure to 1% O_2_ for 24 h. Luciferase activities were normalized by both protein concentration and GFP fluorescence. **c** Wild type and mutant CCNB1 promoter-luciferase constructs were transfected into LLC cells with or without BRG1 followed by exposure to 1% O_2_ for 24 h. Luciferase activities were normalized by both protein concentration and GFP fluorescence. **d** HA-tagged BRG1 and FLAG-tagged E2F1 were transfected into HEK293 cells. Co-immunoprecipitation was performed with indicated antibodies. **e** LLC cells were transfected with siRNA targeting E2F1 or SCR before exposure to 1% O_2_ for 24 h. ChIP assays were performed with anti-BRG1 or IgG. Inset, knockdown efficiency of E2F1. **f** LLC cells were exposed to 1% O_2_ and harvested 24 h later. Re-ChIP assay was performed with indicated antibodies

We next investigated whether BRG1 directly regulates CCNB1 transcription. Reporter assay showed that BRG1 augmented activation of the CCNB1 promoter (−1075) by hypoxia (Fig. 3a). When a series of deletions were introduced, BRG1 was able to activate the CCNB1 promoter until the deletion went beyond −100 (Fig. 3b). Similarly, mutagenesis of a conserved E2F1 site within the CCNB1 promoter abrogated its activation by BRG1 (Fig. 3c), suggesting that BRG1 might rely on E2F1 to activate CCNB1 transcription. Indeed, co-immunoprecipitation showed that BRG1 interacted with E2F1 in cells (Fig. 3d). Further, BRG1 was recruited to the CCNB1 promoter in LLC cells exposed to low oxygen in an E2F1-dependent manner (Fig. 3e). Hypoxia treatment promoted the formation of a BRG1-E2F1 complex on the CCNB1 promoter (Fig. 3f). Combined, these data support a role for BRG1 in regulating hypoxia-induced CCNB1 transcription by interacting with E2F1.

### BRG1 regulates LTBP2 transcription in lung cancer cells

**Fig. 4:**
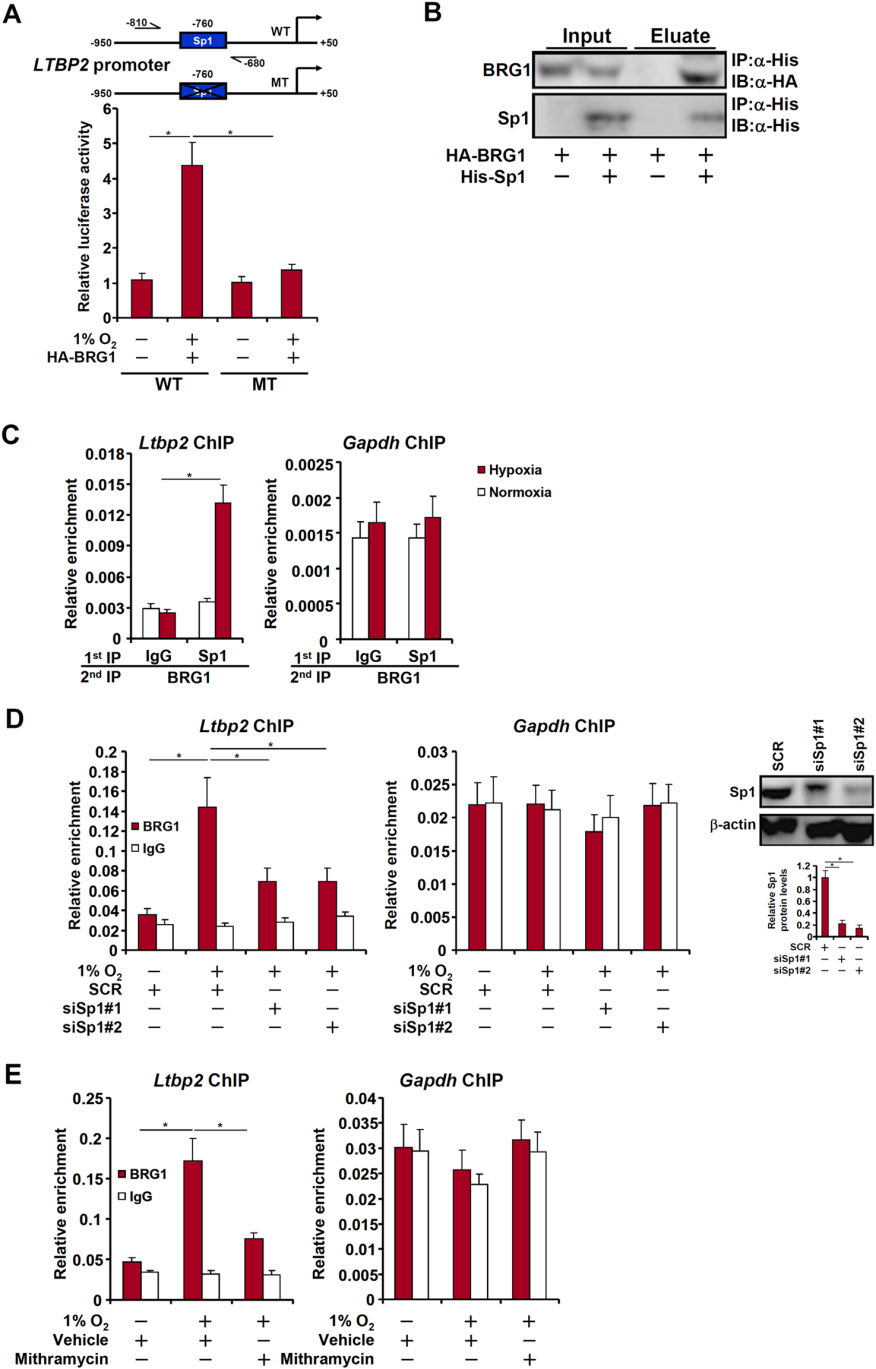
BRG1 regulates LTBP2 transcription in lung cancer cells. **a** Wild type and mutant CCNB1 promoter-luciferase constructs were transfected into LLC cells with or without BRG1 followed by exposure to 1% O_2_ for 24 h. Luciferase activities were normalized by both protein concentration and GFP fluorescence. **b** HA-tagged BRG1 and His-tagged Sp1 were transfected into HEK293 cells. Co-immunoprecipitation was performed with indicated antibodies. **c** LLC cells were exposed to 1% O_2_ and harvested 24 h later. Re-ChIP assay was performed with indicated antibodies. **d** LLC cells were transfected with siRNA targeting Sp1 or SCR before exposure to 1% O_2_ for 24 h. ChIP assays were perormed with anti-BRG1 or IgG. Inset, knockdown efficiency of Sp1. **e** LLC cells were treated with or without mithramycin before exposure to 1% O_2_ for 24 h. ChIP assays were perormed with anti-BRG1 or IgG

We next explored the mechanism whereby BRG1 regulates LTBP2 transcription in LLC cells in response to hypoxia stimulation. BRG1 over-expression enhanced the trans-activation of the LTBP2 promoter in hypoxia-treated LLC cells only when a conserved Sp1 site was left intact (Fig. 4a), suggesting that Sp1 might be necessary for recruiting BRG1 to the LTBP2 promoter. The interaction between Sp1 and BRG1 was confirmed by co-immunoprecipitation assay (Fig. 4b). Further, hypoxia treatment enhanced the interaction between BRG1 and Sp1 on the LTBP2 promoter but not the GAPDH promoter (Fig. 4c). Sp1 silencing by siRNA (Fig. 4d) or inhibition by mithramycin (Fig. 4e) blocked BRG1 recruitment to the LTBP2 promoter. Collectively, these data are consistent with the model wherein BRG1 contributes to hypoxia-induced LTBP2 transcription by interacting with Sp1.

### BRG1 recruits KDM3A to regulate transcription

**Fig. 5:**
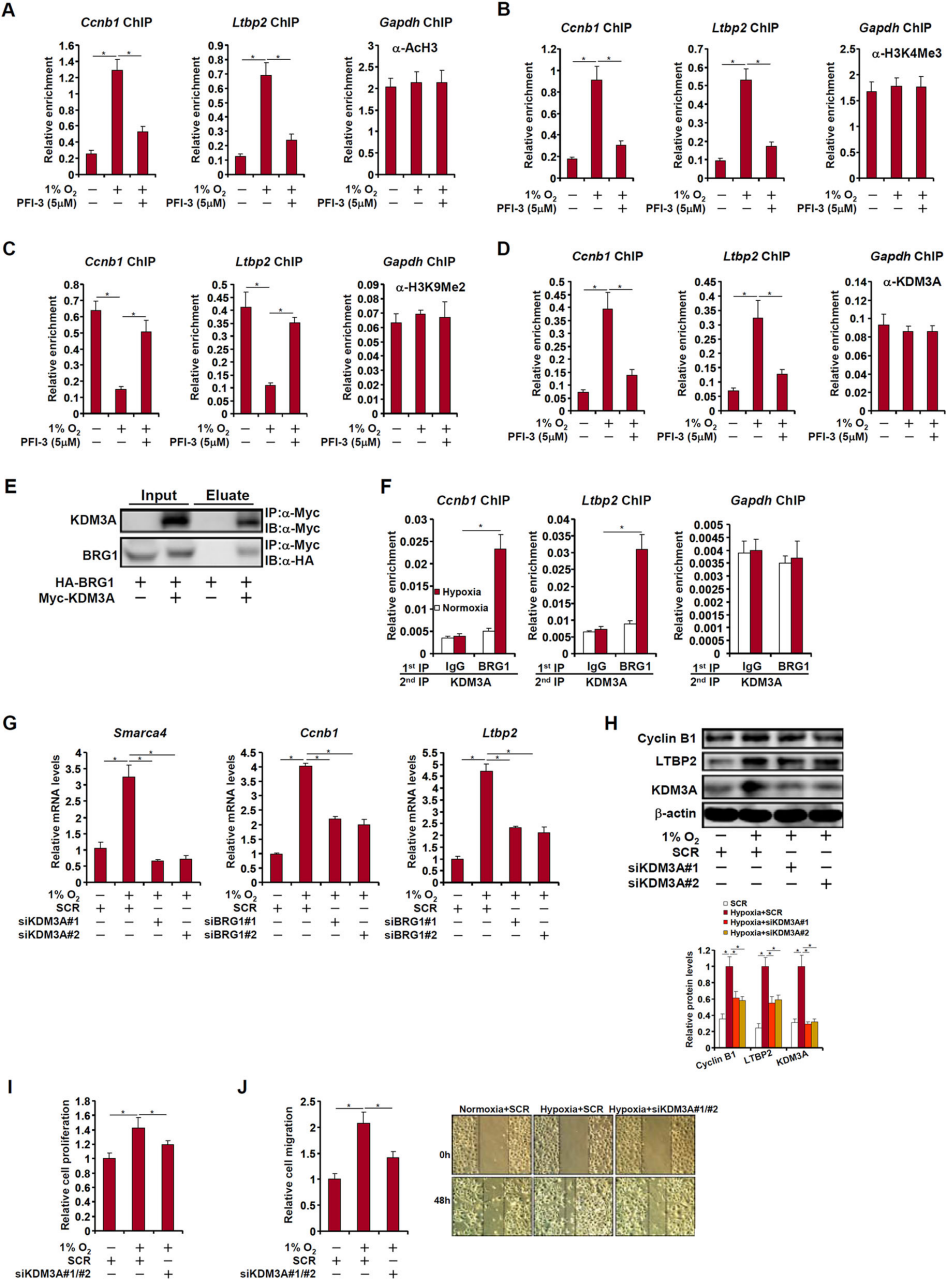
BRG1 recruits KDM3A to regulate transcription. **a**–**d** LLC cells were treated with a BRG1 inhibitor and exposed to 1% O_2_ for 24 h. ChIP assays were performed with indicated antibodies. **e** HA-tagged BRG1 and Myc-tagged KDM3A were transfected into HEK293 cells. Co-immunoprecipitation was performed with indicated antibodies. **f** LLC cells were exposed to 1% O_2_ and harvested 24 h later. Re-ChIP assay was performed with indicated antibodies. **g**–**j** LLC cells were transfected with siRNA targeting KDM3A or SCR before exposure to 1% O_2_ for 24 h. Gene expression levels were examined by qPCR and Western. MTT assay and wound healing assay were performed and quantified as described in Methods

We performed a series of ChIP assays to evaluate the epigenetic mechanism whereby BRG1 regulates the transcription of CCNB1 and LTBP2 in LLC cells. Hypoxia stimulation promoted the accumulation of acetylated histone H3 (Fig. 5a) and trimethylated H3K4 (Fig. 5b) on the proximal CCNB1 and LTBP2 promoters; inhibition of BRG1 by PFI-3 antagonized the enrichment of acetyl H3 and trimethyl H3K4. On the other hand, there was a decrease in dimethyl H3K9 levels on the proximal CCNB1 and LTBP2 promoters, which was abrogated by BRG1 inhibition (Fig. 5c). Concomitantly, hypoxia treatment resulted in increased recruitment of KDM3A, a demethylase specialized in removing dimethyl H3K9, whereas BRG1 inhibition blocked KDM3A recruitment (Fig. 5d). Co-immunoprecipitation confirmed that BRG1 formed a complex with KDM3A in cells (Fig. 5e). More important, Re-ChIP assay demonstrated that stronger BRG1-KDM3A interaction could be detected on the CCNB1 promoter and the LTBP2 promoter following hypoxia stimulation in LLC (Fig. 5f). In addition, KDM3A also contributed to hypoxia-induced CCNB1 and LTBP2 expression in LLC cells (Fig. 5g, h). Functionally, KDM3A appeared to be equivalent to BRG1 in promoting lung cancer cell proliferation (Fig. 5i) and migration (Fig. 5j).

### BRG1 directly activates KDM3A transcription

**Fig. 6:**
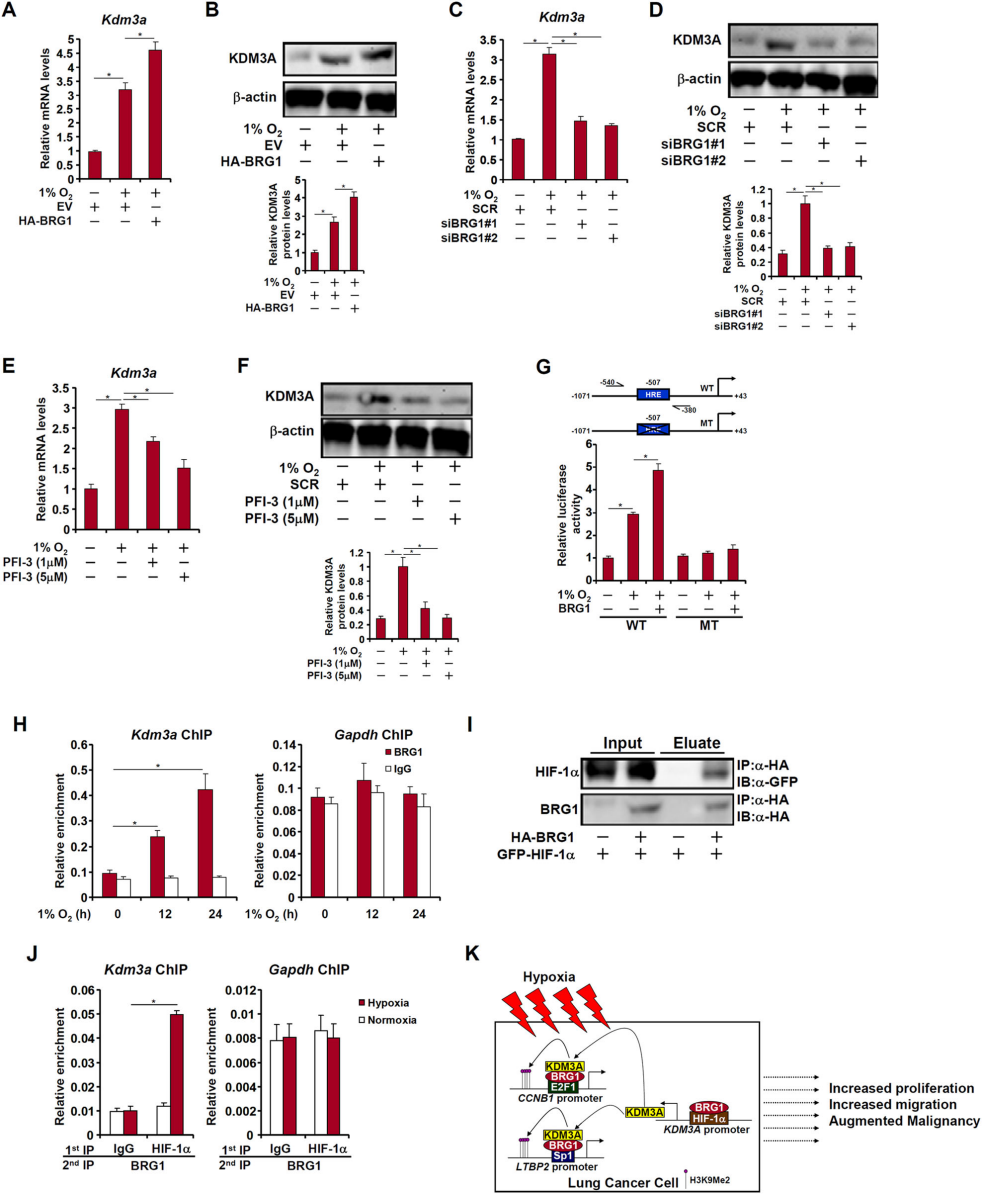
BRG1 directly activates KDM3A transcription. **a**, **b** LLC cells were transfected with HA-tagged BRG1 or an empty vector (EV) before exposure to 1% O_2_ for 24 h. Gene expression levels were examined by qPCR and Western. **c**, **d** LLC cells were transfected with siRNA targeting BRG1 or SCR before exposure to 1% O_2_ for 24 h. Gene expression levels were examined by qPCR and Western. **e**, **f** LLC cells were treated with a BRG1 inhibitor and exposed to 1% O_2_. Gene expression levels were examined by qPCR and Western. **g** Wild type and mutant KDM3A promoter-luciferase constructs were transfected into LLC cells with or without BRG1 followed by exposure to 1% O_2_ for 24 h. Luciferase activities were normalized by both protein concentration and GFP fluorescence. **h** LLC cells were exposed to 1% O_2_ and harvested at indicated time points. ChIP assays were performed with anti-BRG1. **i** HA-tagged BRG1 and GFP-tagged HIF-1α were transfected into HEK293 cells. Co-immunoprecipitation was performed with indicated antibodies. **j** LLC cells were exposed to 1% O_2_ and harvested 24 h later. Re-ChIP assay was performed with indicated antibodies. **k** A schematic model

Previously it has been shown that KDM3A transcription is hypoxia inducible via a conserved HIF-1α site within its proximal promoter^21^. Of note, KDM3A levels were elevated in malignant types of lung cancers paralleling BRG1 up-regulation (Fig. 1h). We therefore sought to determine whether BRG1 might play a role in hypoxia-induced KDM3A transcription. BRG1 over-expression enhanced KDM3A induction by hypoxia (Fig. 6a, b). Conversely, BRG1 depletion (Fig. 6c, d) or BRG1 inhibition (Fig. 6e, f) attenuated KDM3A induction by hypoxia, suggesting that BRG1 might directly control KDM3A levels in LLC cells. To further verify the role of BRG1 in hypoxia-induced KDM3A transcription, a wild-type KDM3A promoter-luciferase construct and a KDM3A promoter construct harboring hypoxia response element (HRE) mutation were transfected into LLC cells. As expected, hypoxia increased the WT KDM3A promoter activity; BRG1 over-expression further enhanced the KDM3A promoter activity. Neither hypoxia nor BRG1 over-expression impacted the MT KDM3A promoter activity (Fig. 6g). ChIP assay confirmed that BRG1 bound to the KDM3A promoter region containing HRE in response to hypoxia (Fig. 6h). The interaction between HIF-1α and BRG1 was further confirmed by co-immunoprecipitation (Fig. 6i) and Re-ChIP (Fig. 6j) assays. Taken together, these data demonstrate that BRG1 directly activates KDM3A transcription by interacting with HIF-1α.

## Discussion

The epigenetic machinery is an integral part of the regulatory network that controls a wide range of pathological processes during cancer oncogenesis and metastasis including proliferation, migration, invasion, angiogenesis, metabolic reprogramming, immune evasion, and resistance to chemotherapy^22^. The effort to investigate the epigenetic mechanism underlying this heinous disease with the ultimate goal of identifying potential therapeutic targets in the past decade has grown exponentially. At the same time, our knowledge and understanding with regard to the epigenetic regulation of lung carcinogenesis has been expanded tremendously especially with the emergence of cancer epigenomics^23^. Here we present evidence to show that expression levels of BRG1, a chromatin remodeling protein, are elevated in human lung cancers as they become more aggressive paralleling the up-regulation of genes involved in cell proliferation (CCNB1) and migration (LTBP2). Further, BRG1 regulates pro-proliferative and pro-migratory transcription in lung cancer cells by interacting with different sequence-specific transcription factors. More important, BRG1 recruits a histone H3K9 demethylase KDM3A to alter the chromatin structure surrounding the target promoters. Finally, BRG1 directly controls the availability of KDM3A by cooperating with HIF-1α to activate KDM3A transcription itself (Fig. 6k). Our data are consistent with recently published reports, which suggest that BRG1 confers growth and metastatic advantages to lung cancers^17,18,24^. Therefore, targeting BRG1 appears to be a viable strategy in the treatment of certain malignant forms of lung cancers.

BRG1 relies on its interactions with sequence-specific transcription factors to become integrated into the transcriptional network that programs cancer malignancy. Here we show that BRG1 interacts with E2F1 to regulate CCNB1 transcription and with Sp1 to regulate LTBP2 transcription. It has been previously suggested that BRG1 primarily functions as a co-repressor for E2F1 in the transcriptional regulation of pro-apoptotic genes such as p14^ARF^ and p73^25^. However, it has been proposed that BRG1 can function either as a co-activator or a co-repressor depending on the specific cell types and cues^26,27^. It is not clear at this point whether the ability to potentiate E2F1-mediated CCNB1 transcription reflects the norm or an exception of the mode of action for BRG1 as an E2F1 co-factor. On the other hand, strong evidence suggests that BRG1 is a key co-activator for Sp1 in the regulation of oncogenesis^28–30^. An open question is whether interactions between BRG1 and factors other than E2F1/Sp1 may contribute to the regulation of lung cancer cell proliferation and migration by BRG1 because we suspect that such complex processes as cell proliferation and migration would depend on the expression of two, instead of a group of, genes (CCNB1 and LTBP2). Clearly, deciphering the BRG1 interactome with ChIP-seq will bring further insights to the understanding of lung cancer oncogenesis.

HIF-1α is the master regulator of cellular response to hypoxia typical to a pro-proliferative and pro-migratory tumor microenvironment. Here we show that BRG1 collaborates with HIF-1α to activate KDM3A transcription. Several known HIF-1α target genes are sensitive to the alteration of BRG1 status although the mechanism is not invariably attributable to an interaction between BRG1 and HIF-1α. Kenneth et al. first reported that BRG1, along with other components of the SWI/SNF complex, is both sufficient and necessary for HIF-1α dependent transcription as assayed by a hypoxia response element (HRE) reporter^31^. Sena et al. have provided additional evidence to show that transcription of a subset of HIF-1a genes relies on BRG1 and/or BRM, the alternative ATPase subunit of the SWI/SNF complex, in hepatocellular carcinoma (HCC) cells^32^. Chen et al. have shown that BRG1 and BRM contributes to hypoxia-induced expression of adhesion molecules in vascular endothelial cells^10^. These findings allude to the possibility that BRG1 may be a de novo co-factor for HIF-1α in modulating the hypoxia response. Several pieces are missing from the jigsaw puzzle of BRG1-mediated regulation of HIF-1α activity. First, the interaction between BRG1 and HIF-1α has yet to be fine-mapped. Second, how BRG1 contributes to HIF-1α dependent transcription on a genomewide scale has not been determined. Finally, the relevance of this BRG1-HIF-1a interplay in lung cancer oncogenesis has not been verified in model animals and in patients. These issues certainly deserve further investigation.

Mounting evidence points to a role for BRG1 in recruiting various histone and DNA modifying enzymes to target promoters to regulate transcription. We report here that BRG1 recruits KDM3A to activate CCNB1 and LTBP2 transcription in lung cancer cells. In addition, KDM3A is elevated in malignant type of human lung cancers and plays an essential role in hypoxia-induced lung cancer cell proliferation and migration. This is in keeping with recent findings that KDM3A promotes oncogenesis of colorectal cancer^33^, prostate cancer^34^, breast cancer^35^, and ovarian cancer^36^. Of interest, BRG1 is necessary for hypoxia-induced KDM3A transcription. Thus, BRG1 controls the KDM3A dynamics by not only modulating its physical association with target promoters but regulating its bioavailability. It would be of great interest to determine whether this BRG1-KDM3A interplay extends beyond the regulation of CCNB1 and LTBP2 transcription and how it contributes to lung cancer oncogenesis in vivo.

In summary, our data as summarized here point to a central role for BRG1 in the epigenetic regulation of lung cancer cell proliferation and migration in vitro. At least two different types of small-molecule BRG1 inhibitors are currently available^37^. Therefore, our data provide renewed rationale for using these chemicals and for exploring novel BRG1-targeting chemicals in clinical trials.

## Materials and methods

### Human lung cancer samples

All human studies were reviewed and approved by the intramural Nanjing Medical University Committee on Ethical Conduct of Studies with Human Subjects. Lung cancer tissues were collected, under informed consent, from surgical resection specimens of patients who had not undergone radiotherapy or chemotherapy in the Affiliated Hospital of Nantong University. Diagnoses of all cases were confirmed by histological examination. Tumor differentiation was graded by the Edmondson grading system. Samples were processed essentially as previously described^38^. Basic patient information is summarized in supplementary Table I.

### Cell culture

The murine lung carcinoma cells (LLC) were authenticated by the Chinese Academy of Sciences Type Culture Collection Cell Bank and were maintained in DMEM (Invitrogen) as previously described^39^. HEK293 cells were purchased from Invitrogen and maintained in DMEM. The cells were re-authenticated using a fingerprint method every 6 months in the laboratory. The last time the cells were authenticated was November 2018. Where indicated, hypoxia (1% O_2_) was achieved by a mixture of ultra-high purity gases (5% CO_2_, 10% H_2_, 85% N_2_) in a 37 °C incubator (Thermo Fisher).

### Plasmids, transient transfection, and reporter assay

HA-tagged BRG1, FLAG-tagged E2F1, His-tagged Sp1, GFP-tagged HIF-1α, Myc-tagged KDM3A, CCNB1 promoter-luciferase constructs, and KDM3A promoter-luciferase constructs have been previously described^21,40–43^. LTBP2 promoter-luciferase construct was made by amplifying ~1 kb of genomic DNA spanning the proximal LTBP2 promoter (−950/+50) and ligating the amplicon into the pGL3 vector (Promega). Mutation constructs were made using the Quickchange Mutagenesis Kit (Agilent). Cells were harvested 48 h after transfection and reporter activity was measured using a luciferase reporter assay system (Promega) as previously described^44–46^. Briefly, cells were plated in 12-well culture dishes (~60,000 cells/well). The next day, equal amounts (0.1 μg) of reporter construct and effector construct were transfected into each well. DNA content was normalized by the addition of an empty vector (pcDNA3). For monitoring transfection efficiency and for normalizing luciferase activity, 0.02 μg of GFP construct was transfected into each well. Luciferase activities were normalized by both protein concentration and GFP fluorescence. Data are expressed as relative luciferase unit compared to the control group arbitrarily set as 1.

### Protein extraction, immunoprecipitation, and western blot

Whole-cell lysates were obtained by re-suspending cell pellets in RIPA buffer (50 mM Tris pH 7.4, 150 mM NaCl, 1% Triton X-100) with freshly added protease inhibitor (Roche) as previously described^47,48^. Specific antibodies or pre-immune IgGs (P.I.I.) were added to and incubated with cell lysates overnight before being absorbed by Protein A/G-plus Agarose beads (Santa Cruz). Precipitated immune complex was released by boiling with 1X SDS electrophoresis sample buffer. Western blot analyses were performed with anti-BRG1 (Santa Cruz, sc-10768), anti-Cyclin B1 (Proteintech, 55004-1), anti-LTBP2 (Sigma, HPA003415), anti-KDM3A (Proteintech, 12835-1), anti-HA (Sigma, H3663), anti-FLAG (Sigma, F3165), anti-His (Invitrogen, MA1-21315), anti-GFP (Proteintech, 50430-2), anti-Myc (Santa Cruz, sc-40), and anti-β-actin (Sigma, A2228) antibodies. All experiments were repeated three times.

### RNA isolation and real-time PCR

RNA was extracted with the RNeasy RNA isolation kit (Qiagen) as previously described^49,50^. Reverse transcriptase reactions were performed using a SuperScript First-strand Synthesis System (Invitrogen). Real-time PCR reactions were performed on an ABI Prism 7500 system with the following primers: human BRG1, 5′-AGTGCTGCTGTTCTGCCAAAT-3′ and 5′-GGCTCGTTGAAGGTTTTCAG-3′; human CCNA2, 5′-TACCTGGACCCAGAAAACCA-3′ and 5′-CACTCACTGGCTTTTCATCTTCT-3′; human CCNB1, 5′-TGTGGATGCAGAAGATGGAG-3′ and 5′-TGGCTCTCATGTTTCCAGTG-3′; human CCND1, 5′-TCCTCTCCAAAATGCCAGAG -3′ and 5′-GGCGGATTGG AAATGAACTT-3′; human CDCA2, 5′- ATGACAGACTTGACCAGAAAGGA-3′ and 5′- CCGACGTTTGGAGGACAACA-3′; human LTBP1, 5′-GCCCTAATGGTGAGTGTTTGA-3′ and 5′- AGATCACAGGGGGATCAGG-3′; human LTBP2, 5′- TGCCCTAGTGGAAAAGGCTA-3′ and 5′-TCACACACTCATCCGCATCT-3′; human KDM3A, 5′-GAGTTCAAGGCTGGGCTATTGT-3′ and 5′-TTCAGCCACTTTGATGCAGCTA-3′; mouse Brg1, 5′-GCACCAAAATCAACGGGAC-3′ and 5′-CTAGGACCCAGCATTG CAC-3′; mouse Ccnb1, 5′-TGCAAACTGTAAGGTTGAAAGC-3′ and 5′-TGTAGAGAGCCAAGTGGAAGG-3′; mouse Ltbp2, 5′′-AAACCCCTCAGCGACCCGCGGCTGC-3′ and 5′′-TGCTTCTGTGAGGACCGGGTGCTCT-3′; mouse Kdm3a, 5′-AAATACGGTTTCGGGATG-3′ and 5′-TACGGGTTTCTCGCTTCT-3′. Data were analyzed by the ΔΔCT method and normalized to 18 s rRNA levels. All experiments were performed in triplicate wells and repeated three times.

### Scratch-wound healing/migration assay

Cells were re-suspended in serum-free media. When the cells reached confluence, scratch wound was created by using a sterile micropipette tip. Cell migration was measured 24 h after the creation of the wound and calculated by Image Pro. Data were expressed as percentage migration compared to control arbitrarily set as 1.

### MTT assay

Cell proliferation was measured using an MTT kit (Abcam) per vendor′s recommendation. Briefly, cells were plated in 12-well plates and allowed to attach overnight before exposure to 1% O_2_ for 24 h. MTT colorimetry was measured before (0 h) and after (24 h) the exposure. Data were expressed as percentage proliferation compared to control arbitrarily set as 1.

### Chromatin Immunoprecipitation

Chromatin immunoprecipitation (ChIP) assays were performed essentially as described before^43,51–60^. In brief, chromatin in control and treated cells were cross-linked with 1% formaldehyde. Cells were incubated in lysis buffer (150 mM NaCl, 25 mM Tris pH 7.5, 1% Triton X-100, 0.1% SDS, 0.5% deoxycholate) supplemented with protease inhibitor tablet and PMSF. DNA was fragmented into ~500 bp pieces using a Branson 250 sonicator. Aliquots of lysates containing 200 μg of protein were used for each immunoprecipitation reaction with anti-BRG1 (Abcam, ab110641), anti-KDM3A (Bethyl Laboratories, A301-538A), anti-trimethyl H3K4 (Millipore, 07-449), anti-dimethyl H3K9 (Millipore, 07-441), anti-acetyl H3 (Millipore, 06-599), anti-E2F1 (Santa Cruz, sc-193), anti-Sp1 (Santa Cruz, sc-14027), anti-HIF-1a (Santa Cruz, sc-10790), or pre-immune IgG. For re-ChIP, immune complexes were eluted with the elution buffer (1% SDS, 100 mM NaCO_3_), diluted with the re-ChIP buffer (1% Triton X-100, 2 mM EDTA, 150 mM NaCl, 20 mM Tris pH 8.1), and subject to immunoprecipitation with a second antibody of interest. Precipitated genomic DNA was amplified by real-time PCR with the following primers: mouse Ccnb1 promoter, 5′-TAAACCTAAGCCCGGCAGAC-3′ and 5′-CCCGATTCGAGAAGACACC-3′; mouse Ltbp2, 5′-ACGCATAACCTCATTCATGTACCGATC-3′ and 5′-AAAGCAATTCTCTAAATTCATAG-3′; mouse Kdm3a promoter, 5′-TCTGTTCCACAAGCATTGACTGG-3′ and 5′-AGGGCTATCACTATTGACACCTGC-3′; mouse Gapdh promoter, 5′-ATCACTGCCACCCAGAAGACTGTGGA-3′ and 5′-CTCATACCAGGAAATGAGCTTGACAAA-3′. A total of 10% of the starting material is also included as the input. Data are then normalized to the input and expressed as % recovery relative the input. All experiments were performed in triplicate wells and repeated three times.

### Statistical analysis

Sample sizes reflected the minimal number needed for statistical significance based on power analysis and prior experience. Two-tailed Student *t* test (for comparison of two groups) or one-way ANOVA with post-hoc Scheffe (for comparison of three or more groups) analyses were performed using an SPSS package. Unless otherwise specified, *P* values < 0.05 were considered statistically significant.

## Supplementary information

online supplementary material
