# Supplementary material for: Epigenetic regulation of lung cancer cell proliferation and migration by the chromatin remodeling protein BRG1
Source: Oncogenesis. 2019 Nov 6;8(11):66. doi: 10.1038/s41389-019-0174-7 (PMC6834663; doi:10.1038/s41389-019-0174-7)
Supplement: Supplementary file 1 — online supplementary material [file 41389_2019_174_MOESM1_ESM.doc]

**Li ZL *et al*: Epigenetic regulation of lung cancer cell proliferation and migration by the chromatin remodeling protein BRG1**

**Online supplementary material**

**Supplementary Table: 1**

**Table I: Clinical characteristics of lung cancer patients**

| **Characteristic** | | **Sample size (n)** |
| --- | --- | --- |
| Patient number | | 18 |
| Mean age (Range) | | 58 (47-72) |
| Gender | Male | 12 |
|  | Female | 5 |
| Smoking history | Smoker | 10 |
|  | Non-smoker | 8 |
| Stage | I&II | 7 |
|  | III&IV | 11 |
| Lymph node | Metastasis | 13 |
|  | Non-metastasis | 5 |
